# Koilocytes indicate a role for human papilloma virus in breast cancer

**DOI:** 10.1038/sj.bjc.6605328

**Published:** 2009-09-22

**Authors:** J S Lawson, W K Glenn, B Heng, Y Ye, B Tran, L Lutze-Mann, N J Whitaker

**Affiliations:** 1 School of Biotechnology and Biomolecular Sciences, University of New South Wales, Sydney, New South Wales, Australia

**Keywords:** breast cancer, HPV, aetiology, expression, HPV E6 oncoprotein, koilocytosis

## Abstract

**Background:**

High-risk human papilloma viruses (HPVs) are candidates as causal viruses in breast cancer. The scientific challenge is to determine whether HPVs are causal and not merely passengers or parasites. Studies of HPV-related koilocytes in breast cancer offer an opportunity to address this crucial issue. Koilocytes are epithelial cells characterised by perinuclear haloes surrounding condensed nuclei and are commonly present in cervical intraepithelial neoplasia. Koilocytosis is accepted as pathognomonic (characteristic of a particular disease) of HPV infection. The aim of this investigation is to determine whether putative koilocytes in normal and malignant breast tissues are because of HPV infection.

**Methods:**

Archival formalin-fixed normal and malignant breast specimens were investigated by histology, *in situ* PCR with confirmation of the findings by standard PCR and sequencing of the products, plus immunohistochemistry to identify HPV E6 oncoproteins.

**Results:**

human papilloma virus-associated koilocytes were present in normal breast skin and lobules and in the breast skin and cancer tissue of patients with ductal carcinoma *in situ* (DCIS) and invasive ductal carcinomas (IDCs).

**Interpretation:**

As koilocytes are known to be the precursors of some HPV-associated cervical cancer, it follows that HPVs may be causally associated with breast cancer.

High-risk human papilloma viruses (HPVs) are candidates as causal viruses in breast cancer (Lawson *et al*, 2006). Human papilloma virus high-risk types 16, 18 and 33 have been identified in breast cancers from 15 widely different populations (Lawson *et al*, 2006). The scientific challenge is to determine whether HPVs are causal and not merely passengers or parasites.

Studies of HPV-related koilocytes in breast cancer offer an opportunity to address this crucial issue. Koilocytes are commonly present in cervical intraepithelial neoplasia. They are epithelial cells characterised by perinuclear haloes (cytoplasmic vacuolation) surrounding condensed nuclei (Ayre, 1951; Koss and Durfee, 1956). Koilocytosis is accepted as pathognomonic (characteristic of a particular disease) of HPV infection (Reid *et al*, 1982). These early findings have been repeatedly confirmed with modern techniques (Roteli-Martins *et al*, 2001; Krawczyk *et al*, 2008). Koilocytosis is a key indicator of subclinical HPV infection and early HPV-associated morphological changes may be used as part of ‘Pap’ screening for cervical cancer (Abadi *et al*, 1998).

Putative (supposed) HPV-associated koilocytes have been observed in some breast neoplasias (de Villiers *et al*, 2005).

There is a biological continuum linking signs of HPV infection leading to koilocytosis, cervical intraepithelial neoplasia and cervical cancer (Reid *et al*, 1984). Forty-eight per cent of women with koilocytosis alone in cervical epithelial cells may develop cervical intraepithelial neoplasia (Evans-Jones *et al*, 1985).

The mechanisms by which high-risk HPV infections cause cervical cancer have been studied in detail (Zur Hausen, 2002). In this study, we have used HPV-associated cervical cancer as a model. High-risk HPV encodes a series of proteins, some of which have oncogenic potential. Human papilloma virus proteins are designated as early (E1–E7) or late (L1 and L2). E5 and E6 act early in transformation (before integration) and are known to disrupt cytokeratin-causing perinuclear cytoplasmic clearing and nuclear enlargement, which leads to the appearance of a koilocyte (Krawczyk *et al*, 2008). E6 and E7 oncoproteins work in concert to disrupt cell cycle regulation and stimulate cell cycle progression by binding and inhibiting the p53 and p110^RB^ tumour suppressor genes, respectively, thereby inducing the proliferation of infected basal cells, which facilitates replication of the viral genome. By binding to and degrading the apoptosis-inducing p53 protein, E6 also inhibits cell death (apoptosis). E7-induced degradation of p110^RB^ often results in a reciprocal overexpression of the cyclin-dependent kinase inhibitor p16^INK4A^, although this is further complicated with increasing cancer stage.

Increasing levels of HPV viral load seem to be associated with an increased risk of developing cervical pre-cancer (Winer *et al*, 2009). This is relevant to HPV-associated breast cancer as the viral load in cervical cancer seems to be over 4000 times higher than in breast cancer (Fiander *et al*, 2007; Khan *et al*, 2008). This huge difference possibly explains the relatively brief time between HPV infection and precancerous changes in the cervix (within 12 months) as compared with the presumed long time between HPV infections and the diagnosis of breast cancer. The extremely low viral load is the probable reason for the considerable difficulty in detection of HPV in breast cancers (Kan *et al*, 2005). Whether koilocytosis is related to breast cancer development is not known.

## 

### Koilocytosis in various HPV-associated cancers and benign lesions.

Putative koilocytes have been identified in HPV 6/11-associated benign laryngeal papillomas (Martins *et al*, 2008), high-risk HPV 16-associated malignant oesophageal lesions (Miller *et al*, 1997), high-risk HPV 11-associated conjunctival papilloma (Minchiotti *et al*, 2006), HPV 6/11-associated benign ductal papillomas of the salivary glands (Haberland-Carrodeguas *et al*, 2003), and high-risk HPV 16 in bladder cancers (Aggarwal *et al*, 2009). These associations between the presence of HPVs and koilocytosis are in addition to their well-established associations in anogenital cancers (Boon and Kok, 1985).

### Pseudo-koilocytes

The diagnosis of koilocytosis has traditionally been based on histological features. Over the years, definitions of koilocytosis have evolved and, as a consequence, excess diagnosis of cervical koilocytosis (positive histological features with negative HPV genetic material) is a problem (Abadi *et al*, 1998). Therefore, investigations of HPV-associated koilocytosis in breast cancer need to include biological assessments in addition to histology.

## Materials and methods

We investigated the possible presence of koilocytes in normal and malignant breast specimens. Archival formalin-fixed invasive ductal carcinoma (IDCs) and ductal carcinoma *in situ* (DCIS) specimens plus normal breast specimens from cosmetic surgery were used.

PCR is by far the most sensitive technique available for the identification of extremely low viral loads, but the technique is associated with problems including both false negatives and false positives, contamination and inconsistent outcomes (Teo and Shaunak, 1995). For this reason, we used both standard and *in situ* PCR together with known positive and negative controls. In addition, we used immunohistochemistry to identify the presence of HPV types 16 and 18 E6 oncoprotein again with both positive and negative controls. We eliminated samples that potentially could give false-positive HPV results, identified by positive *in situ* PCRs without primers. An unknown number of these eliminated specimens would have been true positives. Accordingly, the data cannot be used to make estimates of prevalence of the presence of these viruses.

The methods used for both standard PCR (using DNA extracted from formalin-fixed archival breast cancer specimens) and *in situ* PCR conducted on the same specimens are as described in detail in our companion paper (Heng *et al*, 2009).

### Histology

We based simplified histological definitions of koilocytosis on those established by Reid *et al* (1982) as follows:

1. *Koilocytic cytoplasmic vacuolisation*: perinuclear haloes surrounding cell nuclei.
2. *Koilocytic nuclear change*: pyknosis – nuclear material that may be irregular in size, shape and staining properties. The nucleus is frequently acentric. Binucleation may be apparent. It is now known that ‘binucleation’ involves multilobules caused by HPV-influenced activity at the G2 cell cycle checkpoint (Cho *et al*, 2005). In simple terms, large cells with perinuclear clearing take up a majority of the cells with associated nuclear atypia.

We compared the breast cancer and normal breast specimens that contained koilocytes with cervical cancer specimens that also contained koilocytes.

### Immunohistochemistry

We used standard immunohistochemistry techniques to assess the expression of HPV types 16 and 18 E6 oncoprotein. Human papilloma virus type 16/18 E6 – abCam (ab70) commercial antibodies were used. Human papilloma virus E6 is a nuclear, cytoplasmic and membrane stain. Positive (HPV-positive cervical cancer specimen) and negative (HPV E6 antibody omitted) controls were used.

## Results

Putative (supposed) HPV-associated koilocytes were present in normal breast skin and lobules and in the breast skin and cancer tissue of patients with DCIS and IDCs. These data are shown in Table 1. The identification of koilocytes was determined by the presence of a halo or vacuole in the cell cytoplasm surrounding the nucleus. Pyknosis (intensely stained and irregularly shaped nuclei) was identified in a majority but not all of the putative koilocytes. Some of the specimens were mixed DCIS and IDCs. For reasons of clarity, if invasive characteristics were present, the specimen was classified as IDC.

**Figure 1:**
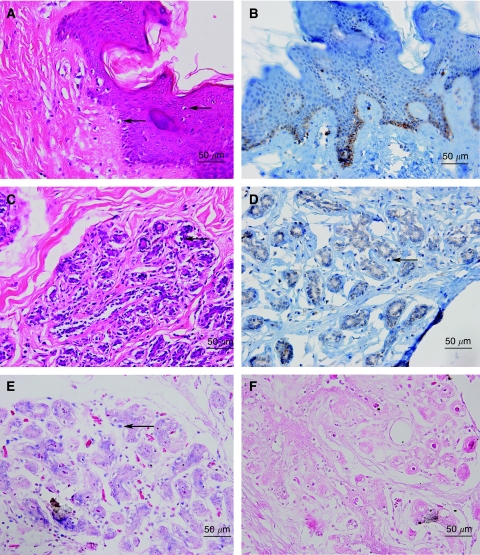
Normal breast specimen. Breast lobules and breast skin showing HPV-associated koilocytes, HPV E6 oncoprotein expression and HPV type 18 by *in situ* PCR in koilocyte nuclei. (**A**) Breast skin with koilocytes (H & E stain), (**B**) breast skin from the same subject showing koilocytes and HPV E6 oncoprotein expression in the basal layers of the skin (immunohistochemistry), (**C**) breast lobules from the same subject with koilocytes (H & E stain), (**D**) breast lobules from the same specimen with HPV E6 oncoprotein expression plus koilocytes (immunohistochemistry), (**E**) positive HPV type 18 expression in the nuclei of koilocytes in the same specimen by *in situ* PCR, (**F**) negative HPV expression in the same specimen by *in situ* PCR with primers omitted (control analysis). The arrows indicate selected putative koilocytes.

**Figure 2:**
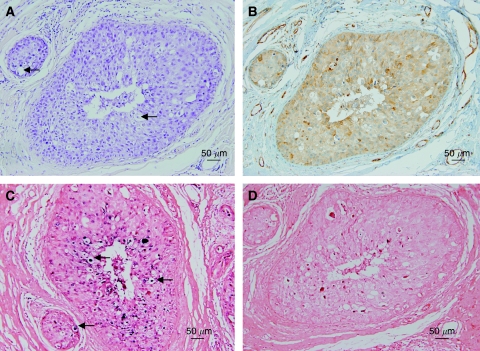
Putative koilocytes in a ductal carcinoma *in situ* breast cancer specimen. (**A**) H & E stain, (**B**) HPV E6 oncoprotein by immunohistochemistry, (**C**) *in situ* PCR for HPV type 18 showing positive staining in the putative koilocytes, (**D**) negative control omitting primers from the *in situ* PCR. The arrows indicate selected putative koilocytes.

**Figure 3:**
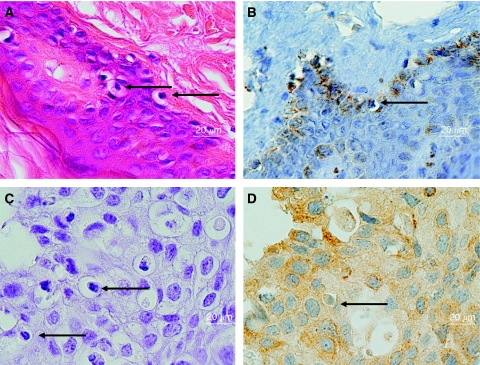
Human papilloma virus (HPV) 16/18 E6 staining in normal and ductal carcinoma *in situ* (DCIS) breast cancer specimens. (**A**) H & E stain of normal breast skin specimen (enlarged version of Figure 1A), (**B**) HPV-E6 oncoprotein by immunohistochemistry of normal breast skin specimen (enlarged version of Figure 1B), (**C**) haematoxylin stain, with eosin omitted, of DCIS specimen (enlarged version of Figure 2A), (**D**) HPV-E6 oncoprotein by immunohistochemistry of DCIS specimen (enlarged version of Figure 2B). The HPV E6 staining (orange colour) in panels B and D appears in the intercellular spaces, some nuclei, cytoplasm and possibly the surface of the cell membranes of the koilocytes. The arrows indicate selected putative koilocytes.

Putative koilocytosis in normal breast lobules and nipple skin of the same subject is shown in Figure 1. This specimen of normal breast tissue was obtained from a normal woman who had cosmetic surgery. Human papilloma virus 18 was identified by *in situ* PCR in the breast lobules and koilocytes of this same specimen. Human papilloma virus oncoprotein E6 is present in the basal layers of breast skin and in the intercellular spaces and cytoplasm of koilocytes, normal breast epithelial cells and malignant cells. There is little E6 nuclear staining. In some specimens stained for HPV E6, there is ‘background’ staining (Figures 1,2,3). The identification of HPV 16 and 18 was confirmed by sequencing the products of standard PCR in a limited number of the specimens. These sequencing data have been reported in the companion paper (Heng *et al*, 2009).

Putative koilocytosis, positive for HPV by *in situ* PCR and HPV E6 oncoprotein in DCIS, is shown in Figure 2 and greatly enlarged in Figure 3.

## Discussion

The presence of HPV-associated koilocytes in normal breast lobules and skin and in DCIS and IDC breast cancer has been shown.

### Validity of the evidence

The viral load of the HPV genome in breast tumours is known from a study based on Japanese subjects in Japan to be extremely low (Khan *et al*, 2008). Although data based solely on Japanese experience cannot be generalised, we and others have had considerable difficulty in the identification of HPV in both fresh and fixed breast cancer specimens from Australian and other Western women (Kan *et al*, 2005). For this reason, we have used both standard and *in situ* PCR plus immunohistochemistry in this investigation. We also used standard criteria for the histological characteristics of koilocytes. We believe the data to be valid because of the confirmation that HPV sequences are present in koilocytes by *in situ* PCR with confirmation by sequencing of the product of standard PCR extracted from the same specimen.

The pattern of HPV E6 staining mainly in the cell cytoplasm and the intercellular spaces is of interest as this same pattern of staining has been observed in cervical tissue lesions (mainly lymphocytes) when stained with the same antibodies (abCam 70) (Kobayashi *et al*, 2002). This adds to the validity of our current observations.

The possible mechanics of HPV transmission in breast cancer is of interest as it is commonly assumed that cell surface-to-surface contact, mainly during sexual activities, is required. It is possible that there may be initial surface-to-surface cell contact with HPV virions that are expressed by desquamating cells during sexual intercourse followed by transmission through the blood or lymphatic systems (Pao *et al*, 1991; Bryan and Brown, 2001). The transmission of HPV infections by oral sexual activities has recently been shown and is also a possible transmission route for HPVs in breast cancer (D’Souza *et al*, 2009). Transmission of high-risk HPVs is also possible without sexual activity as has been shown in families from Finland (Rintala *et al*, 2005).

The mechanics of the specific association between HPV infections and koilocytosis has recently been shown (Krawczyk *et al*, 2008). Koilocytosis may be present as a consequence of infections by both low- and high-risk HPVs. The early HPV proteins designated E5 and E6 cooperate to produce koilocytes in cervical cells.

As the HPV viral load in breast cancer seems to be extremely low, the oncogenic influences of HPVs are likely to be less than in cervical and other anogenital cancers. Therefore, it should not be assumed that the presence of koilocytosis in normal breast tissues commonly leads to breast cancer. Human papilloma virus infections may well be common in the breast, but consequent cancer may be rare.

For the following reasons, we believe it is likely that high-risk HPVs may have an aetiological role in some breast cancers: (i) HPV infections are specifically associated with koilocytosis in cervical and other tissues, (ii) HPVs have been repeatedly identified in breast cancers that have occurred in women from a wide range of populations, (iii) HPV-positive koilocytes are present in normal skin and breast tissues of normal women, (iv) HPV-positive koilocytes are present in many DCIS and some IDC specimens. As HPV-associated koilocytes in the cervix may progress to varying grades of malignancy (Thomison *et al*, 2008), it follows that HPV may be causally associated with breast cancer.

Infections of the cervix by HPV types 16 and 18, as identified in these investigations, are successfully inhibited by the new anti-HPV vaccines (Rambout *et al*, 2007; Koutsky, 2009). It is likely that the same HPV types infecting the breast will also be inhibited by these vaccines and, for the first time, offer primary prevention of some breast cancers.

## Figures and Tables

**Table 1:** The presence of HPV in normal breast, DCIS and IDC specimens

	**HPV by *in situ* PCR**^a^ **(types 16 and 18)**	**HPV by standard PCR**^a^ **(types 16 and 18)**
Normal breast^b^	4/18 (22.2%)	—
DCIS^b^	4/12 (33.3%)	4/10 (40.0%)
IDC^b^	4/9 (44.4%)	3/4 (75.0%)

a HPV identified in normal breast, DCIS and IDC specimens by *in situ* PCR confirmed by standard PCR and sequencing of the product as previously reported (Heng *et al*, 2009).

b The differences in numbers of specimens is because of exclusion of potentially false-positive PCR analyses and poor-quality sections damaged during processing.
